# Shear Wave Elasticity Measurements of Three-Dimensional Cancer Cell Cultures Using Laser Speckle Contrast Imaging

**DOI:** 10.1038/s41598-018-32763-x

**Published:** 2018-09-27

**Authors:** Pei-Yu Chao, Wei-Wen Liu, Shih-Feng You, Pai-Chi Li

**Affiliations:** 1 Graduate Institute of Biomedical Electronics and Bioinformatics, National Taiwan University, Taipei, Taiwan; 2 Department of Electrical Engineering, National Taiwan University, Taipei, Taiwan

## Abstract

Shear wave elastography (SWE) has been widely adopted for clinical *in vivo* imaging of tissue elasticity for disease diagnosis, and this modality can be a valuable tool for *in vitro* mechanobiology studies but its full potential has yet to be explored. Here we present a laser speckle contrast SWE system for noncontact monitoring the spatiotemporal changes of the extracellular matrix (ECM) stiffness in three-dimensional cancer cell culture system while providing submillimeter spatial resolution and temporal resolution of 10 s. The shear modulus measured was found to be strongly correlated with the ECM fiber density in two types of cell culture system (*r* = 0.832 with *P* < 0.001, and *r* = 0.642 with *P* = 0.024 for cell culture systems containing 4 mg/ml Matrigel with 1 mg/ml and 2 mg/ml collagen type I hydrogel, respectively). Cell migration along the stiffness gradient in the cell culture system and an association between cell proliferation and the local ECM stiffness was observed. As the elasticity measurement is performed without the need of exogenous probes, the proposed method can be used to study how the microenvironmental stiffness interacts with cancer cell behaviors without possible adverse effects of the exogenous particles, and could potentially be an effective screening tool when developing new treatment strategies.

## Introduction

Ultrasound elastography has been applied in clinical *in vivo* studies for evaluating the mechanical properties of biological tissue, as changes in the tissue elasticity can be found in various diseases including fibrosis and cancer^1^. Ultrasound shear wave elastography has recently demonstrated that both the mean and the spatial variation of the elasticity of a tumor aid tumor characterization^1–3^. In particular, tumor stiffness was found to be inversely correlated with the percentage of tumor necrosis^2,4^, and the stiffness of the peritumoral stroma was higher than that at the center of a malignant tumor^5^. *In vitro* models have been widely employed for investigating stiffness-mediated cellular functions and behaviors during disease progression. Studies have shown that the stiffening of the cell microenvironment—in particular the extracellular matrix (ECM)—resulted from the increased deposition of collagen through a YAP/TAZ mechanosensor gene self-enhancing loop during tumorigenesis^6,7^, as well as up-regulated expression of the cross-linking enzyme lysyl oxidase promoting the proliferation, invasion and migration of tumor cells^8–11^. In addition, the stiffness of the ECM influences the endothelium that surrounds the tumor. The level of endothelial protein (e.g., CCN1) is increased by the stiffening of the tumor ECM, which subsequently contributes to up-regulation of N-cadherin on the surface of the endothelium, and promotes binding between cancer cells and endothelial cells, which is an important process leading to cancer cell intravasation^12^. Thus, evaluating the dynamics of the mechanical properties of the ECM is important in studies of stiffness-mediated cell behaviors during cancer progression, where the findings could lead to the improvements in cancer treatment strategies.

However, the dynamics of the ECM stiffness during tumor growth have not been well characterized, since the conventional mechanical measurement systems either provide only bulk measurements (i.e., with no spatial information) or subsurface measurements with spatial information about the stiffness distribution in the submillimeter region. The application of shear wave elastography to *in vitro* models has the potential to provide quantitative information on the stiffness distribution of the ECM with millimeter-scale dimensions, and establish linkages between the findings from *in vivo* and *in vitro* studies.

Recent *in vitro* studies in mechanobiology have widely employed three-dimensional (3D) cell culture systems formed from matrix hydrogels, as these assays allow cells to interact with the surrounding cells and matrix in 3D, which more closely mimics the natural environment of the cell^13–15^. However, the dimensions of the 3D cell culture system pose challenges for the mechanical measuring system. Conventional studies of the cell behavior mediated by the stiffness of the ECM were conducted using a substrate with a known stiffness, which is characterized using tensile testing or shear rheometry^16,17^. The stiffness of the substrate can be temporally controlled using techniques such as phototuning polymerization^18^ and enzymatic polymerization or degradation^19,20^. However, the spatial and temporal dynamics of the ECM stiffness resulting from the interactions between the cells and ECM were unknown since the measurement technique requires direct contact, and the stress applied during bulk viscoelasticity measurements may resulted in the destruction of the culture sample.

Particle-tracking microrheology^21,22^ and atomic force microscopy^23–25^ are minimally invasive techniques that have recently been implemented for viscoelastic measurements of 3D cell culture systems with high spatial resolutions (on the micrometer and submicrometer scales, respectively). Particle-tracking microrheology involves imaging and analyzing the motion of embedded fluorescent particles, and with the application of an appropriate rheological model, the viscoelasticity of the sample can be estimated. In contrast, atomic force microscopy does not require the usage of fluorescent particles. It employs a cantilever, which is modeled as an elastic beam with a known elasticity, to indent the sample and the measured deflection of the cantilever is used to estimate the viscoelasticity of the sample. The imaging depth of particle-tracking microrheology is limited by the objective lens used to image the fluorescent particles, whereas atomic force microscopy is limited to surface viscoelasticity measurements. Thus, the dimensions of the 3D cell culture system that can be imaged are restricted in both methods. In addition, each measurement made using atomic force microscopy can only evaluate the viscoelasticity of the sample at a single point, which further limits the temporal resolution as well as the coverage area for viscoelasticity measurement. Brillouin microscopy is a non-invasive and label-free technique that has been recently demonstrated for measuring the bulk modulus of the sample without the need of external loading source^26^. This technique implemented confocal microscopy for detecting the frequency shift in the transmitted laser light caused by thermally generated acoustic phonons^26^. However, measurements in bulk modulus offer lower contrast for evaluating changes in the elasticity of a biological sample, in comparison with shear modulus measurements.

Shear wave imaging is a noninvasive, quantitative elasticity imaging technique in which a time-varying force, generated either by an external vibrator^27^ or by a focused ultrasound beam^28,29^, produces mechanical waves that propagate perpendicularly to the direction of the excitation force (i.e., shear waves). The local shear modulus of the sample can be approximated directly from the local shear wave speed (SWS), which can be measured by tracking the propagation of the shear wave. Ultrasound-based and optical-based shear wave elasticity imaging have recently been applied for monitoring temporal variations in the elasticity of 3D collagen-based cell culture systems^30^ and performing whole-cell elasticity imaging^31^. However, exogenous scatterers are required to be added to the sample in order to produce the speckle pattern necessary for ultrasound imaging to detect the shear wave^30^, and the use of 100× microscope for imaging the propagation of shear waves limits the imaging depth^31^.

Laser speckle contrast shear wave imaging^32,33^ is an optical speckle-based imaging technique for detecting shear waves, which exhibits improved detectability of the presence of motion, spatial resolution, and accuracy of the elasticity imaging, with a trade-off of reduced imaging depth (<20 mm). The high motion sensitivity of laser speckle contrast shear wave elasticity imaging is hypothesized to be a suitable technique for performing elasticity measurements of samples with low scattering properties, such as a 3D cell culture system, with submillimeter spatial resolution and a temporal resolution of 10 s (a shear wave image can be acquired in less than 10 s).

The concentration of the matrix fibers is known to affect the stiffness of the ECM, and this has been used to modulate the stiffness of two-dimensional and 3D culture substrates^34^. However, temporal and spatial tracking of the stiffness of a culture substrate over the culture period and its correlation with the characteristics of the matrix fibers have not been demonstrated. Furthermore, during cancer progression, the behavior of cancer cells invading the surrounding stroma is known to be partially modulated by the mechanical properties of the microenvironment^35,36^. Thus, information about the spatial distribution of the stiffness of the microenvironment on a scale closer to the *in vivo* scenario may increase the understanding of the mechanisms that guide cell migration in response to mechanical stimuli. Hence, in this study we investigated both the temporal and spatial variations in the stiffness of 3D cell culture systems using a laser speckle contrast shear wave imaging system with a total imaging area of 4 mm × 2.2 mm (width × height). The density of the matrix fibers was analyzed using immunofluorescence images, with the results compared with the stiffness measurements. The changes in cell functions such as proliferation and migration due to the temporal and spatial variations in the stiffness of the cell culture sample were investigated.

## Results

### Shear wave speed measurements of the 3D cell culture system using laser speckle contrast shear wave imaging

**Figure 1:**
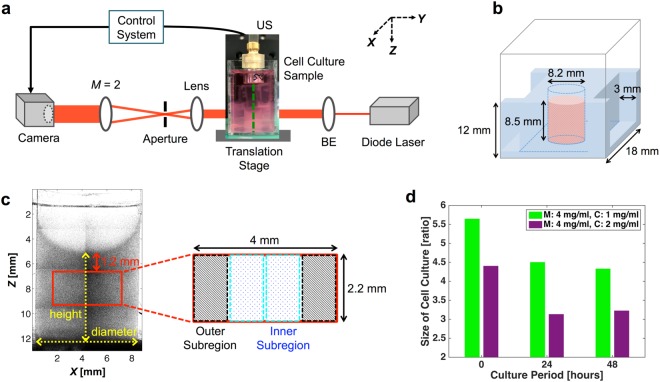
Experimental design. (**a**) Schematic of the imaging system for performing elasticity measurements on a cell culture sample (BE: beam expander, US: ultrasound transducer, *M*: magnification). The green dashed line marks the central plane of the cell culture system imaged using the developed system. (**b**) Schematic of the developed 3D cell culture system. The cell-matrix gel was loaded into a circular hollow in the center of the agarose structure. (**c**) A side view of the cell culture system (in the *X*-*Z* plane), merged from eight optical projection images. The stiffness of the region delineated by the red rectangle is measured by the imaging system. The investigated region can be separated into two inner and two outer subregions. (**d**) Ratio of the sizes of the cell-matrix gel before and after the fixation process for each of the three observation time points (M: Matrigel, C: collagen type I).

The laser speckle contrast shear wave imaging system shown in Fig. 1a was used to image the propagation of the shear wave in the focal plane of the imaging system. The ultrasound transducer used to generate shear wave in the cell culture sample (Fig. 1b) was positioned approximately 15 mm above the cell-matrix gel, which meant that the interrogated region was located approximately 1.2 mm from the top surface of the gel. The location of the interrogated region was selected so as to eliminate the possibility of the shear wave reflecting at the boundary of the cell-matrix gel. The interrogated region comprised four subregions (two inner and two outer subregions), each with dimensions of approximately 1 mm and 2.2 mm in the *X* and *Z* directions, respectively, as shown in Fig. 1c, and the thickness of the subregions (in *Y* direction) is in the scale of submillimeter. The diameter and height of the cell-matrix gel were recorded before shear wave imaging was performed, and the ratio of the dimensions of the cell-matrix gel before and after the fixation process for each observation time-points was calculated. As shown in Fig. 1d, the shrinkage of the cell culture sample due to the fixation process decreased as the culture period progressed. Therefore, to compare the contents of the cell-matrix gel longitudinally, the data shown in Fig. 1d were used to normalize the results from the immunofluorescence analysis and cell counting.

**Figure 2:**
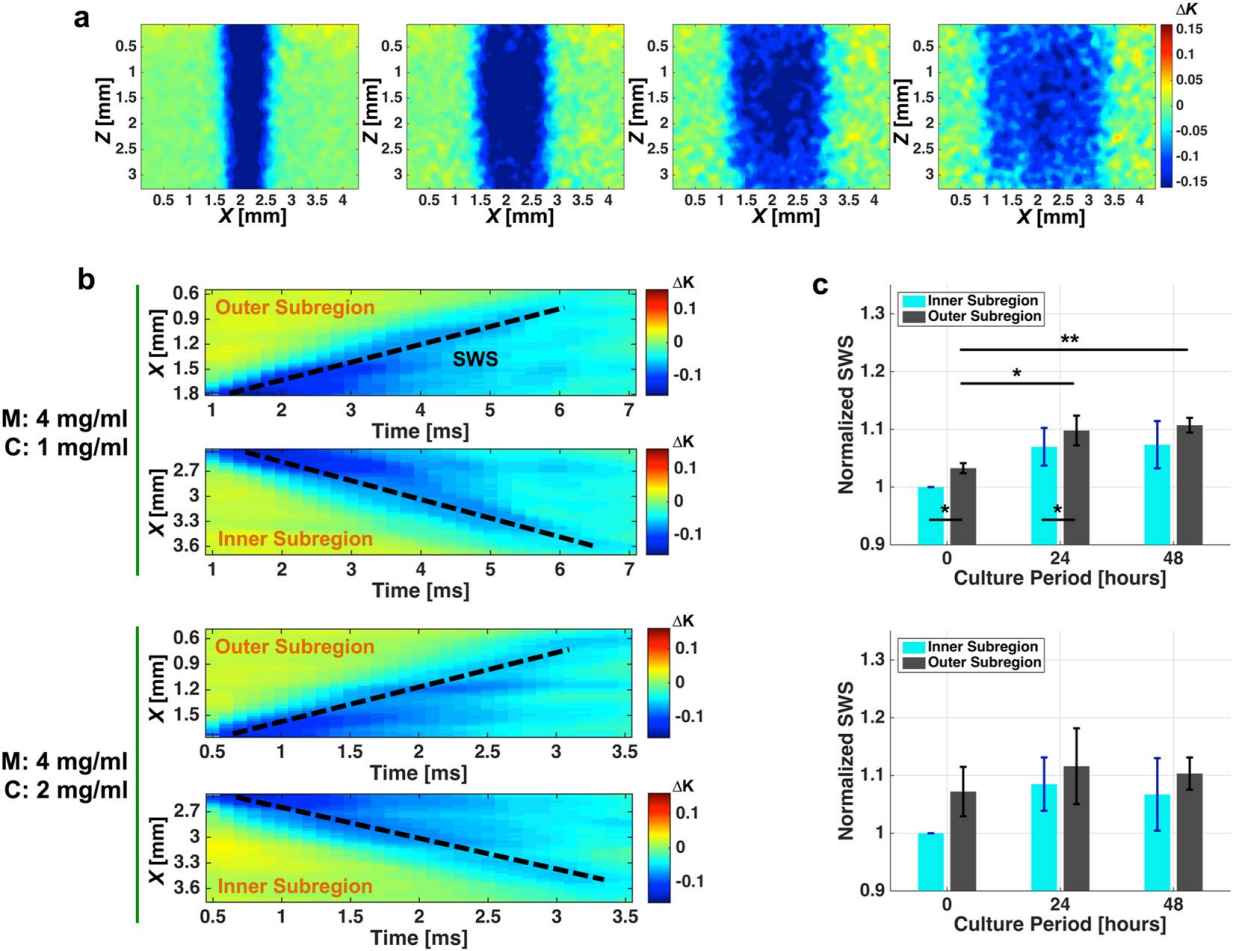
Shear wave imaging of cell culture systems. (**a**) Temporal series of speckle contrast difference maps showing the propagation of the shear wave at 0 ms, 0.5 ms, 1.0 ms and 1.5 ms after the ultrasound transducer delivered an ARF into the cell culture sample. (**b**) Spatiotemporal maps of the inner and outer subregions of the two types of cell culture system (M4C1 and M4C2). The black dashed lines indicate the wavefront slope, which equals SWS. (**c**) Temporal variation of SWS measured for the M4C1 and M4C2 cell culture systems (*N* = 3). *P < 0.05, **P < 0.01.

Figure 2a shows laser speckle contrast images obtained at different time points during the propagation of the shear wave, where the decreased local speckle contrast indicates the location of the shear wave wavefront at 0 ms, 0.5 ms, 1.0 ms, and 1.5 ms after the acoustic radiation force (ARF) had been induced. For each image kernel in the *Z* direction, a spatiotemporal map (Fig. 2b) showing the shear wave propagation distance in the *X* direction versus time can be computed, and SWS is estimated by calculating the slope of this map (shown as the black dashed line in Fig. 2b).

Table 1 lists the SWS values measured for the two types of cell culture system investigated in this study (4 mg/ml Matrigel with 1 mg/ml collagen type I (M4C1) and 4 mg/ml Matrigel with 2 mg/ml collagen type I (M4C2)) for a culture period of 0 hours (defined as the time point after the polymerization process has completed). As indicated in Table 1, SWS corresponding to the initial stiffness of the cell culture sample could be adjusted by using different concentrations of matrix gel.

**Table 1:** SWS measured for two different compositions of cell culture samples mixed with SW480 cancer cells at a culture period of 0 hours.

Composition of the cell culture sample	SWS [m/s]
4 mg/ml Matrigel + 1 mg/ml collagen type I (M4C1)	0.200 ± 0.030
4 mg/ml Matrigel + 2 mg/ml collagen type I (M4C2)	0.335 ± 0.046

The SWS values are mean ± standard deviation values for three independent experiments.

The average SWS within each subregion of the cell culture sample was determined from five repeated measurements. In each longitudinal experiment, the SWS values measured for the cell culture sample were normalized with respect to the value measured for the inner subregion of the cell culture sample with a culture period of 0 hours. As shown in Fig. 2c, the stiffness of the M4C1 cell culture system increased continuously in both the inner and outer subregions. In the M4C2 cell culture system, inner and outer subregions exhibited the same temporal profile of stiffness variation, where the stiffness of the sample increased over the first 24 hours of the culture period and decreased over the subsequent 24 hours. For both cell culture systems, the stiffness was consistently higher in the outer subregion than in the inner subregion.

### Immunofluorescence and hematoxylin-and-eosin staining analysis

**Figure 3:**
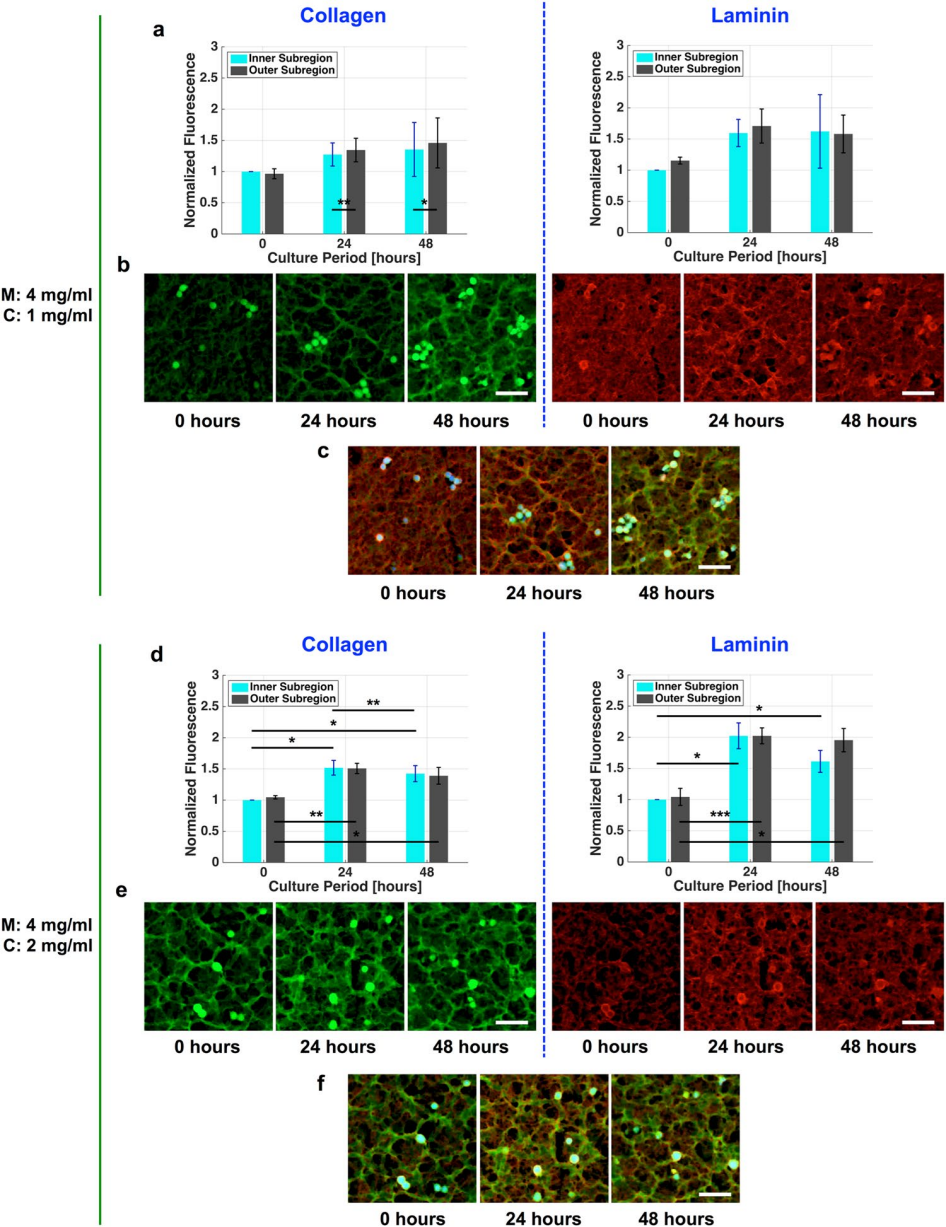
Temporal variations of the fiber density in the cell culture systems. (**a**) and (**d**) Variations in the densities of collagen type I and laminin fibers in the M4C1 and M4C2 cell culture systems, respectively. (**b**) and (**e**) Typical fluorescence images of collagen type I and laminin in the inner subregion for the M4C1 and M4C2 cell culture systems, respectively. (**c**) and (**f**) Overlaid immunofluorescence images of the M4C1 and M4C2 cell culture systems, respectively, where blue fluorescence indicates the nucleus of cancer cells. *P < 0.05, **P < 0.01, ***P < 0.001. Scale bar = 50 µm.

Figure 3a and d show the temporal variations of the density of collagen type I and laminin fibers (which are the main component of Matrigel) during 48 hours of culture for the M4C1 and M4C2 cell culture systems, respectively. The results show that the densities of both collagen type I and laminin fibers increased during 48 hours of culture, which implies that the protein expression are significantly up-regulated over this period. The temporal trend of the fiber density in both cell culture systems resembles the trend observed in the SWS measurements, with slight deviations in the mean density of the laminin fibers in the outer subregion of the M4C1 cell culture system after 48 hours of culture.

Figure 3b and e show fluorescence images of the collagen type I and laminin fibers in the inner subregion of the M4C1 and M4C2 cell culture systems, respectively. Overlaid immunofluorescence images (Fig. 3c and f) show that the laminin deposited in a continuous basement membrane structure surrounding the spheroids in both cell culture systems. In the M4C1 culture system, both collagen type I and laminin fibers were organized into a bundle network after a culture period of 24 hours, while the formation of the bundle networks could be observed after the gelation was completed in the M4C2 culture system. The density of laminin fibers decreased with increasing initial collagen concentration in the cell culture sample after a culture period of 0 hours. Consistent with the normal progression of invasive tumors^37^, these bundles formed tracks perpendicular to the organized basement membrane structure. These features indicate that the increase in the stiffness in the M4C1 cell culture system may have resulted mainly from the bundle formation.

**Figure 4:**
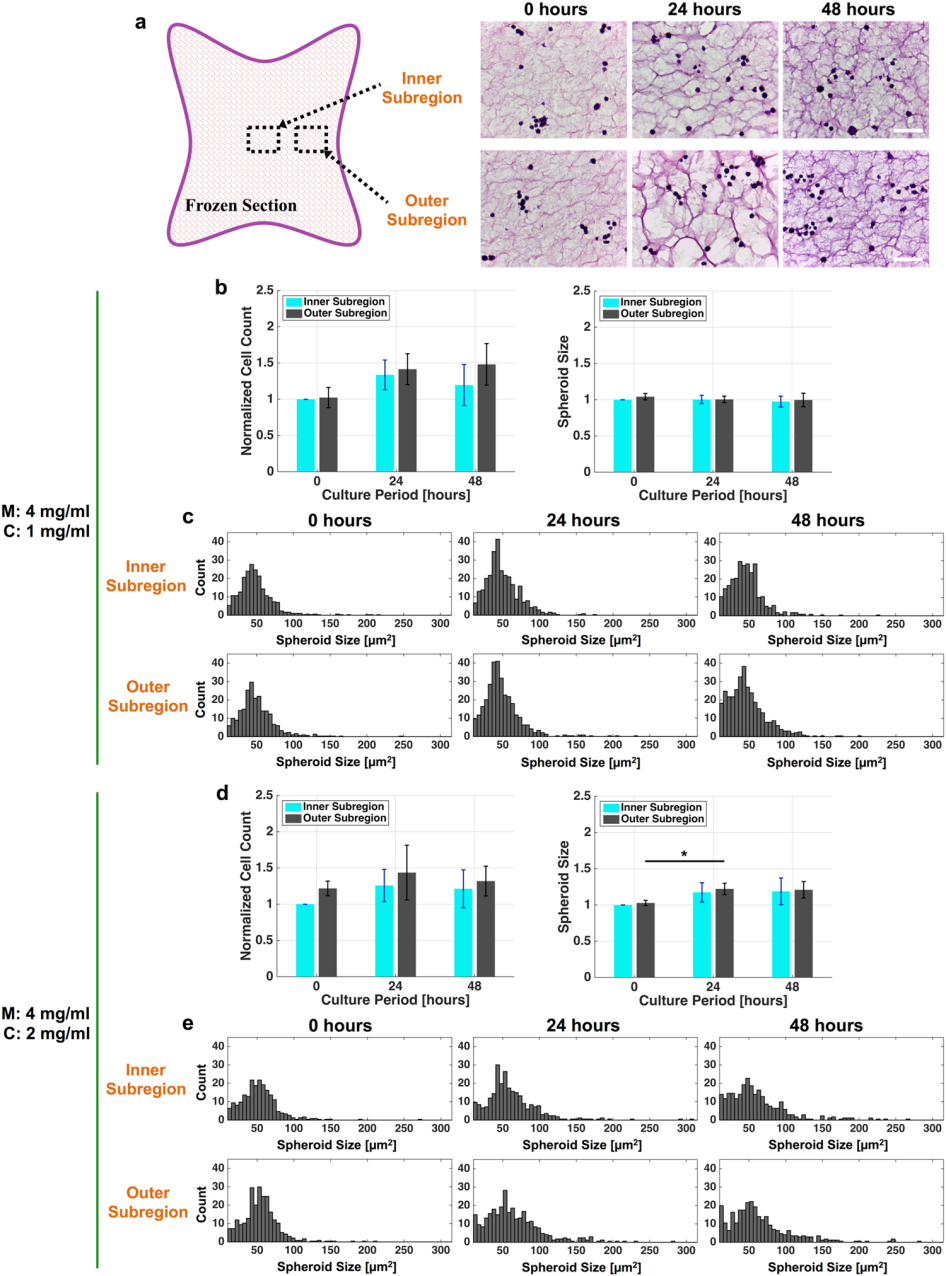
Analysis of H&E stained images. (**a**) Illustration of a frozen section, as well as the approximate locations of the inner and outer subregions in the cell culture sample. H&E stained images of the respective subregions of the M4C1 cell culture sample are also shown. Scale bar = 50 µm. (**b**) and (**d**) Number and size of SW480 spheroids found in the M4C1 and M4C2 cell culture samples, respectively. (**c**) and (**e**) Histograms of the cell counts in the inner and outer subregions of the M4C1 and M4C2 cell culture samples, respectively. *P < 0.05.

Figure 4a illustrates the shape of the frozen section of the cell culture sample and the approximate locations of the inner and outer subregions where the immunofluorescence and hematoxylin and eosin (H&E) stained images were recorded and the fiber density was analyzed (as shown in Fig. 3), as well as the number and size of cultured cells found within these respective subregions. As shown in Fig. 4b and d, the number of cultured cells within the inner subregions had the same temporal trend in both cell culture systems, increasing during the first 24 hours of culture period and then decreasing during the subsequent 24 hours. While the temporal trend of the number of cells found in the outer subregion of the M4C2 follows the same trend as that in the inner subregion, the number of cells found in the outer region of M4C1 continued to increase between 24 and 48 hours of culturing. In both self-made culture systems, the cell viability was successfully maintained and proliferation rates of 1.2- to 1.5-fold were observed during 48 hours of culturing.

Multicellular fusion is one of the fundamental biological processes in normal tissue development that potentiates stem cell plasticity and is associated with cancer metastasis^38–40^. The size of the spheroids was therefore measured to explore the possibility of spheroid fusion under different microenvironmental stiffnesses. The average size of the cultured cells found in the M4C1 cell culture system did not vary significantly either spatially or temporally, whereas that in the M4C2 cell culture system increased with the duration of the culture period. The cell-count histograms in Fig. 4e indicate that the number of spheroids with a cell area greater than 120 µm^2^ increased for the M4C2 cell culture sample during culture periods of 24 hours and 48 hours, which suggests that multicellular spheroid fusion was occurring continuously. In contrast, the size of the spheroids did not increase significantly in the M4C1 culture system, which suggests that fusion tends to occur in stiffer culture systems.

**Figure 5:**
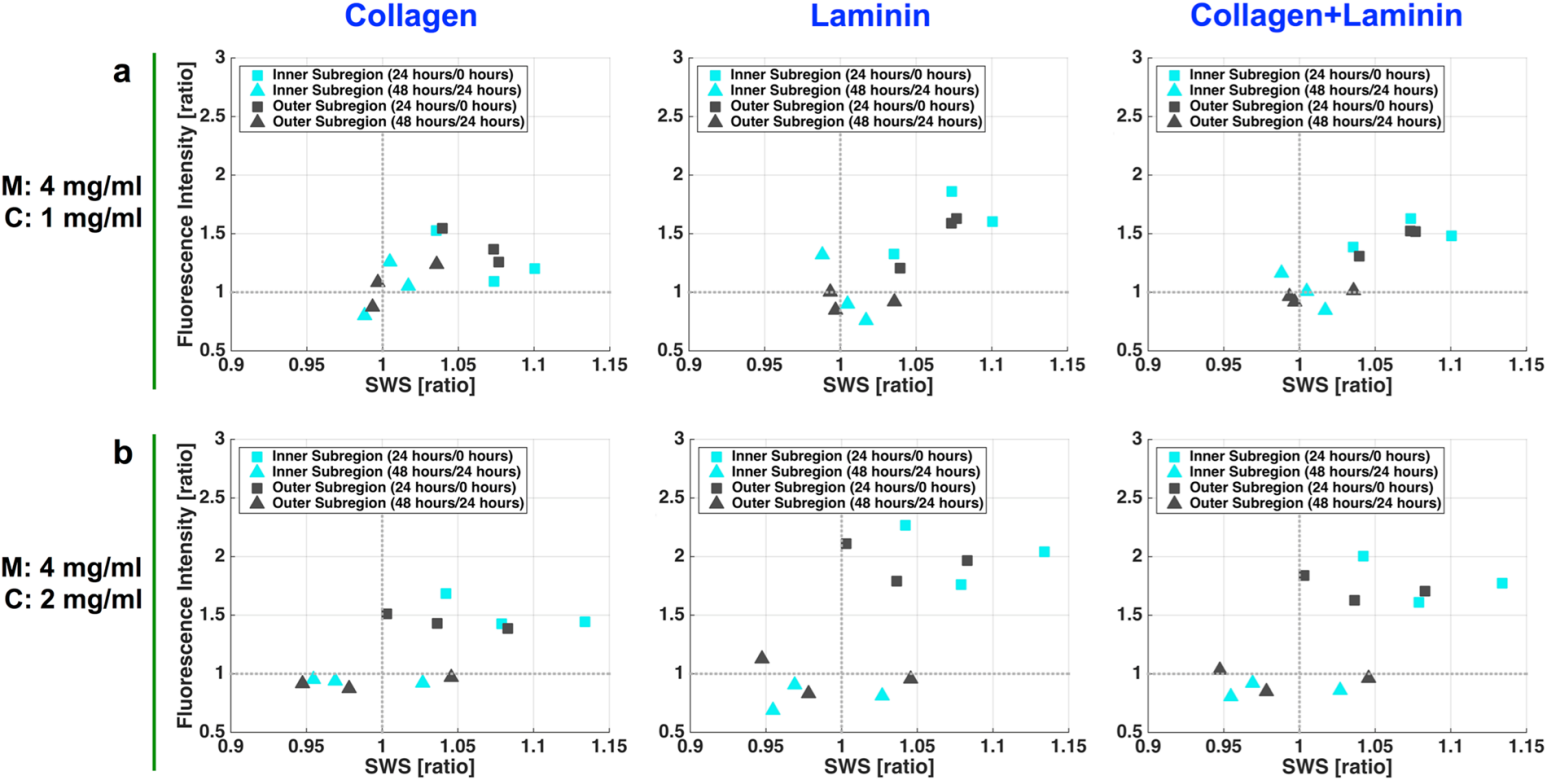
Correlations between SWS measurements and densities of the matrix fibers. Scatter plots of correlations between the ratios of the SWS measurements (i.e., for culture periods of 0–24 and 24–48 hours) and the ratios of the densities of the matrix fibers for the M4C1 and M4C2 cell culture systems, respectively. The 12 data points are from three independent experiments each involving two subregions and two time periods (for culture periods of 0–24 and 24–48 hours).

Figure 5 shows correlation scatter plots of the ratio of the SWS measurements (for culture periods of 0–24 and 24–48 hours) and the ratio of the density of the matrix fibers. For the M4C1 cell culture system, the ratio of the SWS measurements was strongly correlated with the density of laminin fibers (*r* = 0.773, *P* = 0.003), but not with that of collagen type I fibers (*r* = 0.466, *P* = 0.127). For the M4C2 cell culture system, the ratio of the SWS measurements was moderately correlated with the densities of both collagen type I (*r* = 0.630, *P* = 0.028) and laminin (*r* = 0.642, *P* = 0.024) fibers. The concentration of laminin and collagen type I fibers is known to affect the shear modulus of the cell culture system^30,34^. Hence, the combined effect of the matrix fibers was further evaluated. The ratios between collagen type I and laminin matrix were 1:2.4 and 1:1.2 for the M4C1 and M4C2 cell culture systems, respectively. The combined effect of the densities of collagen type I and laminin fibers for the two types of the cell culture system could be defined as

1 $${\rm{M4C1}}={\rm{0.3}}\times {\rm{collagen}}+{\rm{0.7}}\times {\rm{laminin}}$$

2 $${\rm{M4C2}}={\rm{0.45}}\times {\rm{collagen}}+{\rm{0.55}}\times {\rm{laminin}}$$

The density of M4C1 matrix fibers showed a stronger correlation with the ratio of the SWS measurements (*r* = 0.832, *P* < 0.001), whereas the correlation between the density of M4C2 matrix fibers and the ratio of the SWS measurements remained the same (*r* = 0.642, *P* = 0.024).

## Discussion

This study has shown that temporal and spatial changes in the density of matrix fibers are correlated with the local stiffness of the 3D culture substrate. Two types of hydrogel (Matrigel and collagen type I hydrogel) were used in the present self-made 3D cell culture system, and the interaction between the two ECM components (laminin and collagen type I) and stromal stiffness was investigated. Laminin is known to be one of the main components of the basement membrane of a tumor, and collagen type I is considered as the key mediator of stiffness in the tumor microenvironment—this stiffness increased with the formation of the cross-linked collagen bundles, which subsequently promotes tumor invasion. The collagen fibers in a primary tumor tend to be aligned parallel to the tumor basement membrane, whereas in an invasive tumor, the density of the collagen fibers is increased, and stiff collagen bundle structures are formed perpendicular to the tumor basement membrane, to facilitate the migration of cancer cells away from the primary tumor^37,41^. In our results, at least 67% of the acquired data showed a positive correlation (data located in the first and third quadrants of the correlation scatter plot shown in Fig. 5) between the measured SWS and the density of the matrix fibers. Equations 1 and 2 revealed strong correlations (*r* = 0.832 and *r* = 0.642 for the M4C1 and M4C2 cell culture samples, respectively) between the density of the matrix fibers and the measured SWS. These data support the findings that the density of the stromal network was closely associated with the ECM stiffness, not only on the micrometer and submicrometer scales but also on the submillimeter scale.

Rheological measurements using a controlled-strain rheometer (RDA-II, Rheometric Scientific) with a cone and plate geometry (radius of 40 mm, angle of 0.04 rad, and gap of 0.048 mm) was used to verify the accuracy of the imaging system used in this study for measuring the stiffness of the matrix gel. The matrix gel comprising 5 mg/ml Matrigel and 0.12% silica was measured using both systems. Silica was used as the exogenous scatterers for producing the speckle pattern necessary to perform laser speckle contrast shear wave imaging. The storage modulus and loss modulus of the matrix gel measured by the shear rheometer using an oscillating strain of 1% with frequency range of 1–3 Hz were 42.75 Pa and 3.43 Pa, respectively. Assuming that the density of the matrix gel is 1000 kg/m^3^, the group SWS (which is the averaged phase velocity over the frequency range of 1–3 Hz) was estimated at 0.207 m/s. The phase velocity ν is defined as^42^

3 $$\nu (\omega )=\sqrt{\frac{2(G^{\prime} {(\omega )}^{2}+G^{\prime\prime} {(\omega )}^{2})}{\rho (G^{\prime} (\omega )+\sqrt{G^{\prime} {(\omega )}^{2}+G^{\prime\prime} {(\omega )}^{2}})}}$$

where *ρ*, *ω*, *G′* and *G″* are the sample density, angular frequency, storage modulus, and loss modulus measured using the shear rheometer, respectively. The SWS measured using the developed system was 0.193 m/s.

The calculated shear modulus for the 5 mg/ml Matrigel matrix was 42.96 Pa, which is very similar to the measured storage modulus. Hence, Matrigel matrix with a concentration ≤5 mg/ml is considered to be a hydrogel whose low viscosity could be neglected in this study. However, the ratio of the loss modulus over the storage modulus of the collagen type I gel is significantly higher (approximately 1:2)^30^ than that for the Matrigel matrix. Hence, the shear wave phase velocity of the cell culture sample with a higher collagen type I concentration need to be estimated in order to accurately determine the stiffness of the sample. This could explain the weaker correlation between the measured SWS and the density of the matrix fibers for the M4C2 cell culture sample, since the group SWS was measured with the current system setup. The viscosity of the sample can be measured with the imaging system by using amplitude-modulated ultrasound to generate shear waves at different varying frequencies. Tracking the resultant shear waves and determining their SWS values for different frequencies would allow the viscosity of the sample to be estimated^43^.

The stiffness of the culture substrate is known to regulate cell functions. This study found that the changes in the local stiffness of the cell culture sample were associated with changes in the number of cultured cells found within the region of observation. This suggests that an increased elasticity of the matrix promotes the proliferation of SW480 cancer cells. In addition, the spatial variation in SWS of the cell culture system—that is the stiffness gradient present in the cell culture system with a higher SWS in the outer subregion than the inner subregion throughout the entire duration of the culture process—may promote SW480 cancer cells to migrate from the inner subregion toward the outer subregion. The action of durotaxis^44^ is observed as the ratio between the numbers of SW480 cancer cells found in the outer and inner subregion increasing with the culture period for the M4C1 cell culture sample.

The components of the cell culture system, namely the cancer cells and ECM can be used as natural light scatterers to produce the speckle patterns necessary for shear wave measurements when the cell culture system is illuminated with a coherent light source. This allows the imaging system to perform noninvasive, noncontact elasticity measurements on the cell culture system without the need for exogenous probes. Furthermore, the stiffness in regions with dimensions on the millimeter scale can be imaged and measured within an imaging acquisition time of less than 15 minutes. This temporal resolution of the imaging system may increase its suitability for large-scale investigations of cell migration.

## Materials and Methods

### Cell culture system

The cell culture system consisted of an agarose structure and cell-matrix gel. The agarose structure with agarose mass concentration of 0.65% (SeaKem® LE Agarose) was designed to support the cell-matrix gel during polymerization and the subsequent culture process. In the preparation for cell culturing, the agarose structure was immersed in 4.6 ml of culture medium (Dulbecco’s Modified Eagle Medium, Gibco^TM^, with nutrient mixture F-12 and 10% fetal bovine serum) and incubated for 24 hours. Prior to the preparation of the cell-matrix gel, the culture medium was removed and the portion of the glass at the bottom of the circular hollow of the agarose structure was coated with 0.1 mg/ml poly-D-lysine.

The matrix gel comprised a mixture of Matrigel (Corning) matrix and collagen type I (Corning). Two types of cell culture system with different concentrations of collagen type I were used: 4 mg/ml Matrigel with either 1 mg/ml or 2 mg/ml collagen type I. The matrix gel was prepared by first mixing collagen type I with a pH neutralizing buffer containing 1 M NaOH, PBS, 0.9 mM CaCl_2_, 0.5 mM MgCl_2_, and sterilized distilled water. Matrigel matrix was added and thoroughly mixed with the collagen type I mixture before the cell suspension (2.35 × 10^6^ cells/ml in culture medium) was added to the matrix gel mixture. The cell-matrix gel was prepared on ice in order to avoid polymerization. SW480 colon cancer cells were obtained from ATCC.

The cell-matrix gel was carefully loaded into the circular hollow of the agarose structure, and two side channels of the agarose structure were filled with 200 µl of culture medium to temporarily supply nutrients to the cultured cells. The entire cell culture sample was placed in a humidified chamber and allowed to polymerize in an incubator at 37 °C and 5% CO_2_ for 120 minutes. The polymerized cell culture sample was filled with 5.5 ml of culture medium, which was replaced daily, and kept incubated until shear wave imaging was performed.

### Shear wave elasticity measurements

Shear wave elasticity measurements were performed using a system that we developed; the details of the imaging technique and postprocessing algorithms are available elsewhere^33^. In brief, the system consisted of a transmission optical imaging system, where a 50 mW, 785 nm coherent light source (OBIS, Coherent) is used to illuminate the cell culture sample. Imaging lenses in a 4-*f* configuration with a magnification of twofold were used to image the speckle pattern of the cell culture sample onto a CCD camera (Manta G-145B NIR, Allied Vision Technology). An in-house single-element 18 MHz focused ultrasound transducer with focal length of 17.5 mm and an aperture diameter of 7 mm was used to induce an ARF, which resulted in the generation of shear waves in the cell culture sample. A driving sequence of 2,000 cycles of sinusoidal pulses was used to induce an ARF in the cell culture sample. Since the acoustic axis was perpendicular to the optical axis, the shear wave propagating in the *X* direction was imaged. The disturbances induced by the ARF and the propagating shear wave changed the phase of the transmitted optical wave, which resulted in local spatial blurring of the imaged speckle pattern. The speckle contrast (∆*K*) was computed to evaluate the spatial blurring caused by the acoustic disturbances and subsequently used for visualizing the shear wave wavefront. The speckle contrast is defined as

4 $${\rm{\Delta }}K={(\frac{{\sigma }_{s}}{\langle I\rangle })}_{\mathrm{US}\mathrm{On}}-{(\frac{{\sigma }_{s}}{\langle I\rangle })}_{\mathrm{US}\mathrm{Off}}$$

where *σ*_*s*_ and $\langle I\rangle$ are the standard deviation and mean intensity of pixel values within a image kernel, and US On and US Off denoted the speckle image captured when the ultrasound transducer is in nonactivate and activate state. Stroboscopic imaging was adopted for imaging the shear wave propagation at effective frame rates of 10,000 frames/s and 5,000 frame/s for the M4C2 and M4C1 cell culture samples, respectively. SWS within an interrogated region of the cell culture sample was calculated by computing the shear wave propagation distance versus time to produce the spatiotemporal map, where SWS equals the slope of this map and was estimated by applying least-squares linear fitting to the arrival time point of the shear wave wavefront for each propagation distance kernel; that is the lowest speckle contrast difference observed at each distance kernel in the *X* direction. Typically, the time required to perform shear wave elasticity imaging on a cell culture sample, which included five repeated shear wave measurements for each of the four interrogated subregions within the sample, is approximately 15 to 20 minutes.

### Immunofluorescence, hematoxylin and eosin staining

Immediately after shear wave measurements have been performed, the cell culture sample was fixed in 4% paraformaldehyde and 25% glutaraldehyde (Sigma-Aldrich) at a ratio of 24:1 at room temperature for 20 hours. Frozen sections with a thickness of 10 µm obtained from the cell culture sample was stained with collagen type I antibody (GeneTex), laminin antibody (abcam), and Hoechst 33258 (ThermoFisher Scientific). Histological investigation of the sections was using H&E for nuclear and cytoplasmic labeling, respectively. The immunofluorescence of the cell culture samples was imaged using a fluorescence microscope with a 20× objective (AX70, Olympus), whereas the H&E stained samples were imaged using a bright-field microscope with 10× and 40× objectives (DM500, Leica).

### Fiber density calculation

ImageJ software (NIH) was used to calculate the densities of collagen and laminin fibers. Image regions containing only the matrix fibers were manually selected, each comprising at least 850,000 pixels. The fiber density was defined in this study as

5 $${\rm{Fiber}}\,{\rm{density}}=\frac{{\sum }_{i}^{N}{I}_{i}-B}{N-P}$$

where *I* is the fluorescence intensity of pixel *i* (measured as an 8-bit number), *B* is the background fluorescence intensity, *N* is the total number of pixels selected, and *P* is the total number of background pixels.

### Cell count and size distribution analysis

ImageJ software was used to analyze the number and size of the cancer cells found in H&E stained images within a region with dimensions of 1.02 mm × 1.18 mm (width × height). A standard particle analysis algorithm was used.

### Statistical analysis

MATLAB (MathWorks) was used to produce all of the graphs and perform the statistical analysis. Data are presented as mean and standard deviation values. The two-tailed paired Student’s *t*-test was used to identify significant differences (*P* < 0.05) between longitudinal measurements.

## Data Availability

Original data are available from the authors upon request.
